# Comparative Study of Surface-Active Properties and Antimicrobial Activities of Disaccharide Monoesters

**DOI:** 10.1371/journal.pone.0114845

**Published:** 2014-12-22

**Authors:** Xi Zhang, Fei Song, Maierhaba Taxipalati, Wei Wei, Fengqin Feng

**Affiliations:** 1 College of Biosystems Engineering and Food Science, Zhejiang University, Hangzhou, Zhejiang, China; 2 Yunnan Collage of Traditional Chinese Medicine, Kunming, Yunnan, China; 3 Zhejiang Key Laboratory for Agro-Food Processing, Zhejiang University, Hangzhou, Zhejiang, China; 4 Beijing Institute of Nutrition, Synutra International Inc., Beijing, China; 5 Fuli Institute of Food Science, Zhejiang University, Hangzhou, Zhejiang, China; National Central University, Taiwan

## Abstract

The objective of this research was to determine the effect of sugar or fatty acid in sugar ester compounds on the surface-active properties and antimicrobial activities of these compounds. Disaccharides of medium-chain fatty acid monoesters were synthesized through transesterifications by immobilized lipase (Lipozyme TLIM) to yield nine monoesters for subsequent study. Their antimicrobial activities were investigated using three pathogenic microorganisms: *Staphylococcus aureus*, *Escherichia coli* O157:H7 and *Candida albicans*. Their surface-active properties including air–water surface tension, critical micelle concentration, and foaming and emulsion power and stability were also studied. The results showed that all of the tested monoesters were more effective against *Staphylococcus aureus* (Gram-positive bacterium) than against *Escherichia coli* O157:H7 (Gram-negative bacterium). The results demonstrated that the carbon chain length was the most important factor influencing the surface properties, whereas degree of esterification and hydrophilic groups showed little effect.

## Introduction

Fatty acid sugar esters are receiving increasing attention as odorless, nontoxic and biodegradable nonionic surfactants, which are mild to the skin. Sucrose fatty acid esters have certification of GRAS FDA 21CFR 172.859, and could be used as food additives. They are interested by the food industry because they possess many attractive properties, such as emulsification, emulsion stabilization, foaming. For example, sugar esters include a wide range of hydrophilic–lipophilic balance (HLB) values from 1 to 16 achieved with different degrees of esterification, and these esters are commonly employed in the food industry as emulsifying agents [1]–[3]. Other fields of application include pharmaceuticals, detergents, cosmetics and pesticides as a result of their excellent surface and antimicrobial properties [1], [4]–[7]. Previous research has revealed that disaccharide medium–chain fatty acid monoesters display significant activity against several food and clinical isolates. Habulin et al have reported that sucrose monolaurate could inhibit *Bacillus cereus* at concentration of 9.375 mg/mL. [6] Ferrer et al measured the effects of lauroylsucrose and lauroylmaltose against *Bacillus sp*. and *Escherichia coli*. [7] Surfactants may influence cell membranes at low concentration, which could lead to change the permeability of cell membrane, [8] with subsequent metabolic inhibition, growth arrest or cell lysis [9].

Fatty acid sugar esters can be synthesized chemically and enzymatically by interesterification and transesterification. Compared with the chemical synthesis, enzymatic synthesis yields light-colored products with fewer isomers and with limited byproducts [10], [11]. Several lipases have been studied for the synthesis of sugar esters in recent years, like *Candida antarctica* lipase, *Mucor miehei* lipase, *Pseudomonas* sp. lipase and *Thermomyces lanuginosus* lipase [12]–[15]. Lipozyme TLIM is a commercial immobilized *T. lanuginosus* lipase with high selectivity that is regiospecific for specific hydroxyl groups (6-OH for sucrose and 6′-OH for maltose), which could be used for the synthesis of regioselectivity monoesters [11].

In the present investigation, we assessed the surface properties (including air–water surface tension, CMC, and foaming and emulsion power and stability) and antimicrobial activities of nine monoesters: sucrose monolaurate (SL), maltose monolaurate (ML), lactose monolaurate (LL); sucrose monodecanoate (SD), maltose monodecanoate (MD), lactose monodecanoate (LD); and sucrose monooctanoate (SO), maltose monooctanoate (MO), lactose monooctanoate (LO). Many literatures of sugar esters have been reported in the recent years, however, few systematic studies of medium-chain fatty acid monoesters (C8-C12) and the structure–function relationships of these molecules have been reported.

## Materials and Methods

### Chemicals

Lipozyme TLIM was purchased from Novo Nordisk (Denmark). Molecular sieves (4 Å), 2-methylbutanol, dimethylsulfoxide (DMSO), sucrose, maltose, lactose and *n*-hexane were from Sinopharm (China). Raffinose pentahydrate was from Alfa Aesar (France). Vinyl laurate and 6-*O*-monolaurate were from Sigma (Denmark). Vinyl octanoate and vinyl decanoate were purchased from TCI (Japan). Ryoto sucrose ester (L1695) was supplied by Mitsubishi-Kasei Food Corporation (Japan). All the reagents and solvents were of analytical grade. Vinyl fatty acid esters, 2-methyl-2-butanol and DMSO were stored over molecular sieves (4 Å), at least 24 h prior to use. Double-distilled water with a surface tension equal to 69.3 mN/m at 25°C was used in all experiments.

### Microorganisms

*Staphylococcus aureus* CICC 21600 and *Escherichia coli* O157:H7 CICC21530 were provided by China Center of Industrial Culture Collection, Beijing, China, and were grown and maintained in nutrient broth and on nutrient agar (Hangzhou Microbiological Agents Co. Ltd, China). *Candida albicans* CMCC (B) 98001 was provided by the Institute of Microbiology, Chinese Academy of Sciences, Beijing, China, and maintained in Sabouraud broth medium (SDB) and on Sabouraud agar medium (Hangzhou Microbiological Agents Co. Ltd, China). Strains were maintained at 4°C. Cultures were transferred to tryptic soy broth (TSB) or SDB and incubated at 37°C for 18 h for bacteria, or 30°C for 36 h for fungus, respectively, to obtain a working culture.

### Enzymatic synthesis of disaccharides of monolaurate, monodecanoate and monooctanoate

Sugar esters (or biosurfactants) were synthesized by transesterification reactions. The experiments were conducted in flasks by adding sucrose, maltose or lactose (0.04 mmol); Lipozyme TLIM (1 g); molecular sieves (1 g); 2-methylbutanol (8 mL); DMSO (2 mL); and vinyl ester (0.4 mmol). Then, the mixture was magnetically stirred at 50°C and 200 rpm for 4 h. The products of transesterification were determined by thin-layer chromatography, high-performance liquid chromatography and mass spectroscopy.

### Thin-layer chromatography

Thin-layer chromatography was performed on silica gel plates using chloroform–methanol–acetic acid–water (78∶20∶2∶0.2 v/v/v/v) as the eluting system. The compounds were colored by spraying a color agent with *p-*anisaldehyde–acetic acid–95% ethanol–sulfuric acid (9.2∶3.75∶338∶12.5 v/v/v/v) and visualized by heating at 105°C for 15 min.

### High-performance liquid chromatography

The concentrations of the sugar esters were quantified by high-performance liquid chromatography using a Waters pump (Waters 1525) with refractive index detector (Waters 2414). A Purospher RP-18e column (5 µm×250 mm×4.0 mm^2^, Merck) was used, and the mobile phase was a mixture of methanol and water (85∶15 v/v).

### Extraction and refining of the products

At the end of the reactions, the immobilized lipases together with molecular sieves were removed by filtration. The 2-methyl-2-butanol was evaporated by vacuum distillation and then *n*-hexane (1∶1 v/v) was used to extract the residual vinyl ester. The oil phase (containing vinyl ester) was discarded and aqueous phase (containing DMSO) was mixed with saturated sodium chloride solution (1∶1 v/v) and then two volumes of butanone to extract the sugar esters. The butanone containing the sugar esters was evaporated to obtain the crude product.

Column chromatography on silica gel (300–400 mesh) was used to separate the monoester, diester and sugar. The elution phase was chloroform–methanol (80∶20 v/v), and monitoring was by thin-layer chromatography.

### HLB calculation

According to Griffin, the HLB values of nonionic surfactants can be calculated using the following formula:
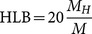
where *M*_H_ is the molar mass of the hydrophilic moiety and *M* is that of the whole surfactant molecule [16].

### Air–water surface tension *γ*_a/w_

Wilhelmy plate method was used to measure surface tensions of sugar esters in aqueous solutions according to force measurements at 25°C, which is similar to Soultani et al [17].

### CMC and *γ*_CMC_ evaluation

CMC values of the sugar esters in aqueous solutions were calculated from the breaking point in *γ*_a/w_ versus log_10_ concentration plots at 25°C. The parameter *γ*_CMC_ is the surface tension corresponding to the CMC [17].

### Foamability and foaming stability

Aqueous solutions of the sugar esters (10 mL) of different concentrations from 0.1 g/L to 0.5 g/L were placed in 50 mL tubes, and the height of each solution (*H*_0_, cm) was measured. Then, each solution was mixed using a homogenizer at 13 500 rpm for 2 min and the foam height (*H*_2_, cm) and the total height (*H*_1_, cm) were determined immediately. After standing for 10 min, 20 min, 30 min, 40 min and 50 min, the foam height (*H*_3_, cm) was recorded at 25°C. All the experiments replicated three times. The foamability and foaming stability were calculated using the following equations:
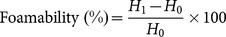





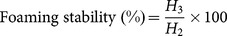



### Emulsion power and stability

Monoester solutions (10 mL) at a concentration of 0.02% (w/v) and soybean oil (10 mL) were placed in 50 mL tubes, and homogenized at 8000 rpm for 2 min to mix two phases, then stood for 10 min to measure the height of the emulsion layer (*H*_1_, cm). After 30 min, 1 h, 1.5 h, 2 h and 24 h, the height of the emulsion layer (*H*_2_, cm) was measured at 25°C. The initial height (*H*_0_, cm) of solutions and soybean oil was also measured. All the experiments replicated three times. The emulsifying ability and emulsion stability were calculated using the following equations:
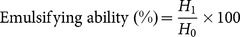





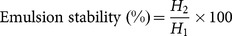



### Antimicrobial activity assay

The minimum inhibitory concentrations (MICs) of the sugar esters were determined using broth microdilution assay [8], [18], [19]. Appropriate quantities of sugar esters were added to broth (pH 6.5±0.5 for bacteria or 5.5±0.5 for yeast), yielding final concentrations of 32, 63, 125, 250, 500, 1000, 2000 and 4000 µg/mL. The corresponding dilutions were inoculated with a suspension of the test organisms on TSB or SDB to a final concentration of 10^4^ CFU/mL. The volumes of sugar ester solution and bacterium suspension were 100 µL. There were three kinds of controls for the test: (i) blank: uninoculated TSB media during the experiment; (ii) negative control: uninoculated TSB media only containing the sugar esters; (iii) positive control: inoculated TSB medium without sugar ester. The experiments were conducted in three replicates. The 96-well plates were incubated for 24 h at 37°C or 48 h at 30°C, and the optical density (OD) at 595 nm for 0 and 24 h of the culture was measured with a Multiskan MK3 microplate reader (Thermo Labsystems, Finland). MICs at 24 h were defined as the lowest concentration at which the bacterial growth was completely inhibited (| OD | <0.05). MIC ≥4000 µg/mL was defined as no antimicrobial activity [20].

### Statistical Analysis

According to the homogeneity of variance of data, Duncan's multiple range test and Games-Howell test were used to determine the significance of difference within treatments for each treatment, 3 replicates were performed and the mean values were calculated. Statistical analysis was run with a confidence level of 95% (p<0.05). All statistical analyses were performed using SPSS statistic software (Version 20.0 for Mac).

## Results and Discussion

### Enzymatic synthesis of sugar fatty acid esters and purification

In order to obtain higher yields of monoester and reduce byproducts, the reaction conditions were conducive for the synthesis of the monoester. Vinyl esters were chosen as acyl donors because the rate of transesterification of sugar and vinyl esters was much faster than with alkyl esters [21]. During the reaction process, the formed vinyl alcohol tautomerized to a low-boiling-point acetaldehyde, which was conducive to the transesterification. According to previous work, the best solvent was *tert-*butanol-DMSO (4∶1 v/v), and monoester content accounted for 70%, while there was a very low percentage of diester (<5%) [15]. Thus, this medium was chosen for the synthesis of the nine monoesters: SL, ML, LL; SD, MD, LD; and SO, MO, LO.

The purity of each monoester was higher than 90% after refining with liquid-liquid extraction and column chromatography, and the side-products were diesters. The products were used for further research.

### Surface-active properties

In colloidal and surface chemistry, CMC is defined as the concentration of surfactants above which micelles form and all additional surfactants added to the system form micelles [22]. CMC is an important characteristic parameter for evaluating the activity of a surfactant.

**Figure 1:**
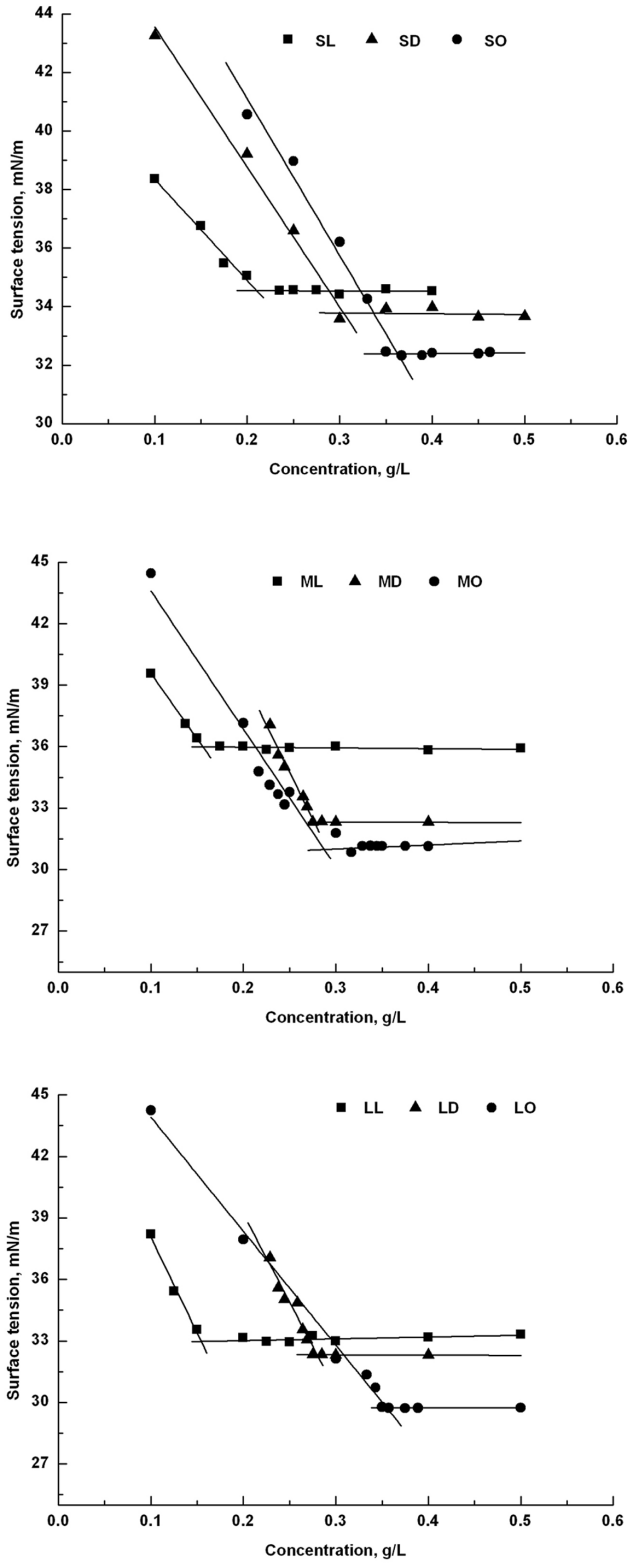
Surface tension versus concentration plots for sugar monoesters.

Fig. 1 shows the surface tension data for sucrose, maltose and lactose monoesters. There is a turning point on every curve at surface tension point, which indicated that the monoester could migrate together to the liquid interface extremely fast at that point [23]. Values of *γ*_CMC_ of monocaprylates were lower than those of monolaurates, showing that the ability of the former to reduce the water surface tension in aqueous solution was better than that of the latter. Values of *γ*_CMC_ decreased and CMC increased when the carbon chain length decreased. These results were similar to those of previous work [24]. Soultani [17] and Ducret [25] reported that a lower hydrophobicity sugar ester with higher CMC always possessed a stronger ability to reduce the surface tension. Some disaccharide fatty acid monoesters with carbon chain lengths from C12 to C16 have been measured to support the viewpoint. [11], [15], [24], [26] Table 1 summarizes HLB, CMC and *γ*_CMC_ of the monoesters and a commercial sucrose lauroyl monoester (L-1695) at 25°C. L-1695 is a mixture of different degrees of esterification of lauroyl sucrose with 80% of 6- and 6′-monoester and 20% of sucrose di- and triesters. The CMC and *γ*_CMC_ of L-1695 were lower than those of SL, which could indicate that monomer aggregation occurred at much lower concentrations in the presence of diesters, which has been explained as the molecular interaction between mono- and diesters (bridging micelle mechanism, surface competition and change of solubility) [23], [27].

**Table 1:** Values of HLB, CMC, *γ*_CMC_, Γ, *A*, Δ*G* estimated for sugar esters.

Surfactant	HLB	CMC (mM)	*γ*_CMC_ (m/Nm)	Γ×10^−5^ (mol/m^2^)	*A* (Å^2^)	Δ*G* (kJ/mol)
**SL**	13.1	0.45	34.54	0.91	18.25	−29.05
**ML**	13.1	0.32	35.97	1.65	10.06	−30.44
**LL**	13.1	0.31	33.06	1.03	16.12	−29.94
**L-1695**	12.4	0.42	32.96	1.30	12.77	−27.89
**SD**	13.8	0.60	33.78	1.76	9.43	−28.32
**MD**	13.8	0.56	32.33	1.97	8.43	−28.47
**LD**	13.8	0.56	31.59	1.85	8.97	−28.47
**SO**	14.5	0.78	32.36	1.17	14.19	−27.68
**MO**	14.5	0.66	31.15	1.01	16.44	−28.16
**LO**	14.5	0.76	29.73	1.15	14.44	−27.73

Surface excess (Γ in mol/m^2^), area per molecule (*A* in Å^2^) and Gibbs free energy of adsorption (Δ*G* in kJ/mol) have been estimated from the surface tension curves, and are given in Table 1 [17], [28], [29].

The surface excess is the extra amount per unit area of the solute that is present at or near the surface when the surface is equilibrated with the mobile phase containing the solute. The equation for the calculation of the surface excess is
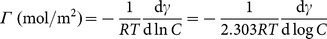
where *R* is the gas constant (8.31 J/(mol K)); *T* the temperature (K); *γ* the surface tension (N/m); and *C* the concentration of surfactant (mol/L).

The area per molecule (*A*) represents the mean area available to each molecule forming monolayers. The area of an adsorbed molecule (in Å^2^ per molecule) at the surface can be calculated from the surface excess using the following formula:
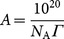
where *N*_A_ is the Avogadro constant (6.023×10^23^/mol).

As the CMC is known, the Gibbs adsorption energy of a sugar ester molecule at the liquid surface can be calculated using
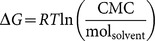



For an aqueous solution, the water molarity can be used for the calculation:
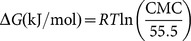



The values of Γ and *A* were little different among all the sugar esters because the hydrophilic groups were disaccharides with similar polarity. Δ*G* was related to the length of the hydrophobic chain and concentration of adsorption equilibrium, so Δ*G* changed accordingly. These values are relatively low compared with those of other literature because they depend strongly on the fitting of experimental data.

### Foamability and foam stability

The foamability and foam stability for different concentrations of aqueous solution of sugar monoesters were measured at 25°C, and the results are shown in Table 2. The foamability varied with the concentration of monoesters, the hydrophobic moiety chain length and the hydrophilic saccharide group. The foaming power and stability rose observably (*p*<0.05) as the concentration of sugar esters increased from 0.1 to 0.5 g/L. Because the increasing concentration of sugar monoester could improve the viscosity of the solution, the liquid could not exude from the bubble film easily and reducing the speed of the film becoming thin, thus delaying film rupturing [30].

**Table 2:** Foamability (%) of different esters at different concentrations^a^.

Surfactant	0.1 g/L	0.2 g/L	0.3 g/L	0.4 g/L	0.5 g/L
**SL**	22.7±3.4^Dab^	38.9±2.4^Cb^	42.3±1.6^Cd^	76.4±4.0^Bbc^	87.7±4.0^Ab^
**ML**	18.9±2.7^Dab^	28.7±3.0^Cc^	81.3±3.0^Ba^	88.5±4.0^Bab^	91.7±4.2^Ab^
**LL**	22.1±0.9^Da^	63.3±3.8^Ca^	91.5±2.5^Ba^	97.8±2.0^Ba^	117.3±1.9^Aa^
**L-1695**	19.2±1.6^Dab^	32.2±2.9^Cbc^	54.2±2.1^Bc^	79.5±1.5^Abc^	80.1±1.3^Ac^
**SD**	15.9±4.1^Bab^	30.2±5.0^ABbc^	32.1±1.5^Ab^	36.1±1.0^Ad^	36.5±1.6^Ade^
**MD**	18.8±2.3^Eb^	36.1±6.9^Dbc^	57.3±8.1^Cbcd^	73.1±2.4^Bc^	92.45±3.1^Ab^
**LD**	17.4±1.2^Eb^	26.4±3.3^Dc^	33.3±3.4^Cbd^	44.3±2.0^Be^	57.3±7.1^Acd^
**SO**	0.0±0.0^Dc^	3.5±1.2^Dde^	7.6±1.2^Ce^	21.5±4.1^Bf^	31.94±2.2^Ae^
**MO**	0.0±0.0^Dc^	6.8±1.0^Cd^	20.8±0.0^Bf^	18.4±2.7^Bf^	37.5±4.6^Ae^
**LO**	0.0±0.0^Dc^	2.43±0.9^Ce^	14.6±1.3^Bg^	17.4±1.1^Bf^	22.0±1.3^Af^

a Values in each group with different letters represent significant difference (*p* <0.05). Superscript upper-case letters in the same row indicate comparison with different concentrations of the same surfactant. Superscript lower-case letters in the same column indicate comparison with different surfactants with the same concentration.

LL showed the best foamability, its value being five-fold greater than that of LO at 0.5 g/L. Overall, foaming power of lauryl monoesters was better than that of decanoyl and capryloyl esters (*p*<0.05), which indicated that the foam height increased with an increase of hydrophobic moiety chain length for medium-chain fatty acid monoesters. Moreover, the level of foam varied with the degree of esterification. The foaming power of L-1695 was obviously lower than that of SL because the foaming ability of a diester is weaker than that of a monoester. Husband showed that pure sucrose monolaurates had better foaming properties than pure sucrose dilaurates [27]. The foam heights of maltose and lactose fatty acid monoesters were higher than those of sucrose fatty acid monoesters, as the hydrophilic group influenced the foamability. Sucrose is composed of a pyranoside (glucose) and a furanoside (fructose), while maltose and lactose are each formed from two pyranosides (two glucoses for maltose, and galactose and glucose for lactose). The different foaming abilities of disaccharide monoesters with the same acyl chain could be due to their various compositions.

**Figure 2:**
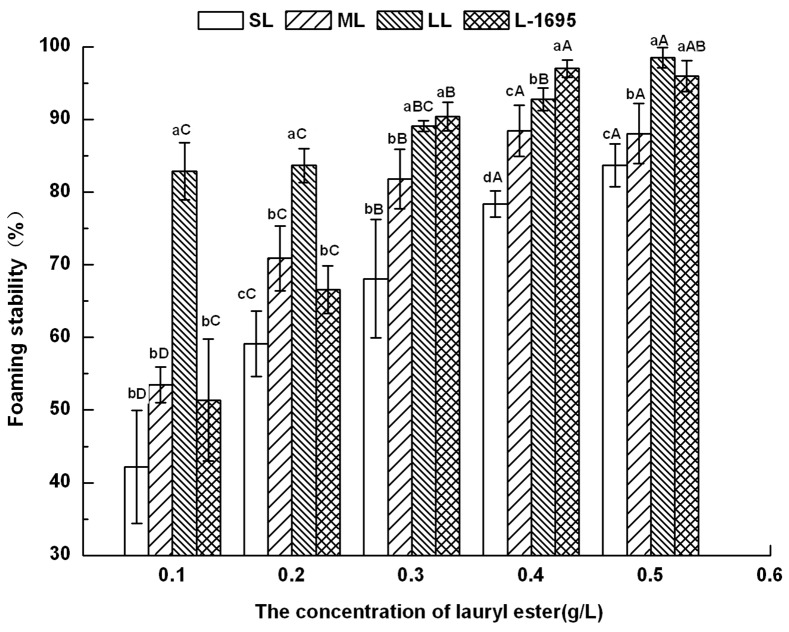
Foaming stability of different concentrations of lauryl monoesters for 50 min at 25°C. Upper-case letters indicate comparison with different concentrations of the same surfactant. Lower-case letters indicate comparison with all surfactants with the same concentration.

For lauroyl esters, foam could be maintained for all concentrations of aqueous solution for 50 min (shown in Fig. 2), while the foam stability of decanoyl esters could be measured at high concentration above 0.4 g/L for only 30 min, which did not exceed 70%. Furthermore, the foam stability of capryloyl esters was even worse than that of decanoyl esters, as foam collapsed within 10 min at all concentrations. Kempen showed similar result that the foam of oligofrutose decanoyl monoester started to coarsen during foam formation and oligofrutose lauroyl showed better foamability and stability than former one [31]. It is demonstrated that the hydrophobic group was an important factor influencing foaming. Probably because the surface-adsorbed molecules of sugar ester with longer carbon chain had an enhanced interaction with each other, the foam power was increased [30]. Esters with fatty acid chain lengths between C10 and C16 had a low initial surface tension and a low surface tension at equilibrium showed excellent foamability and stability because they were able to quickly migrate to the interface to form small bubbles with a long half-life time [31]. L-1695 displayed better foam stability than SL because the addition of diester to monoester improved its foaming properties at low concentrations [27].

**Figure 3:**
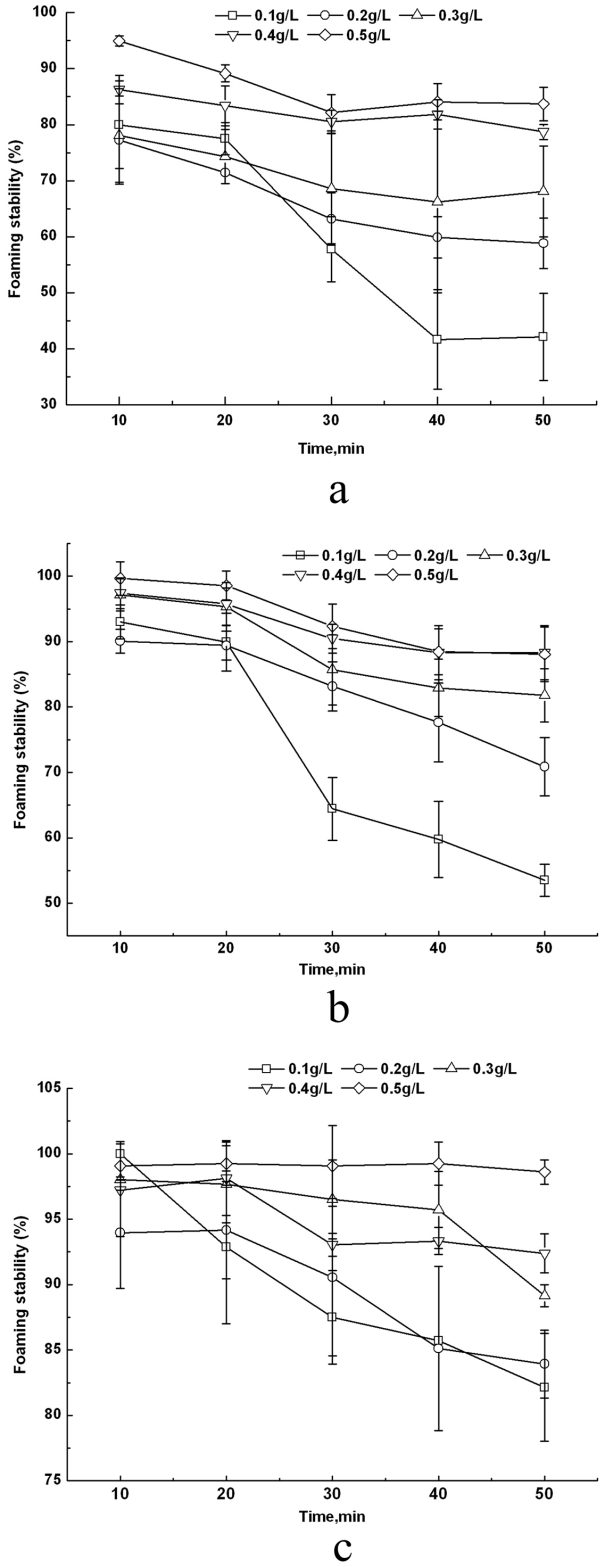
The foaming stability of sugar monolaurates of different concentrations and standing times: (a) SL; (b) ML; (c) LL.

The foaming stability was influenced by the interaction of concentration of monoester aqueous solution and standing time. Fig. 3 shows the foam stability of lauroyl esters decreased during standing time from 10 min to 50 min. The decreasing level was greater for lower concentration, especially after 20 min. LL displayed the best foaming stability, as the foam height dropped slowly. Foaming stability of sugar monolaurates did not change significantly at concentrations over 0.4 g/L.

### Emulsifying ability and emulsion stability

**Figure 4:**
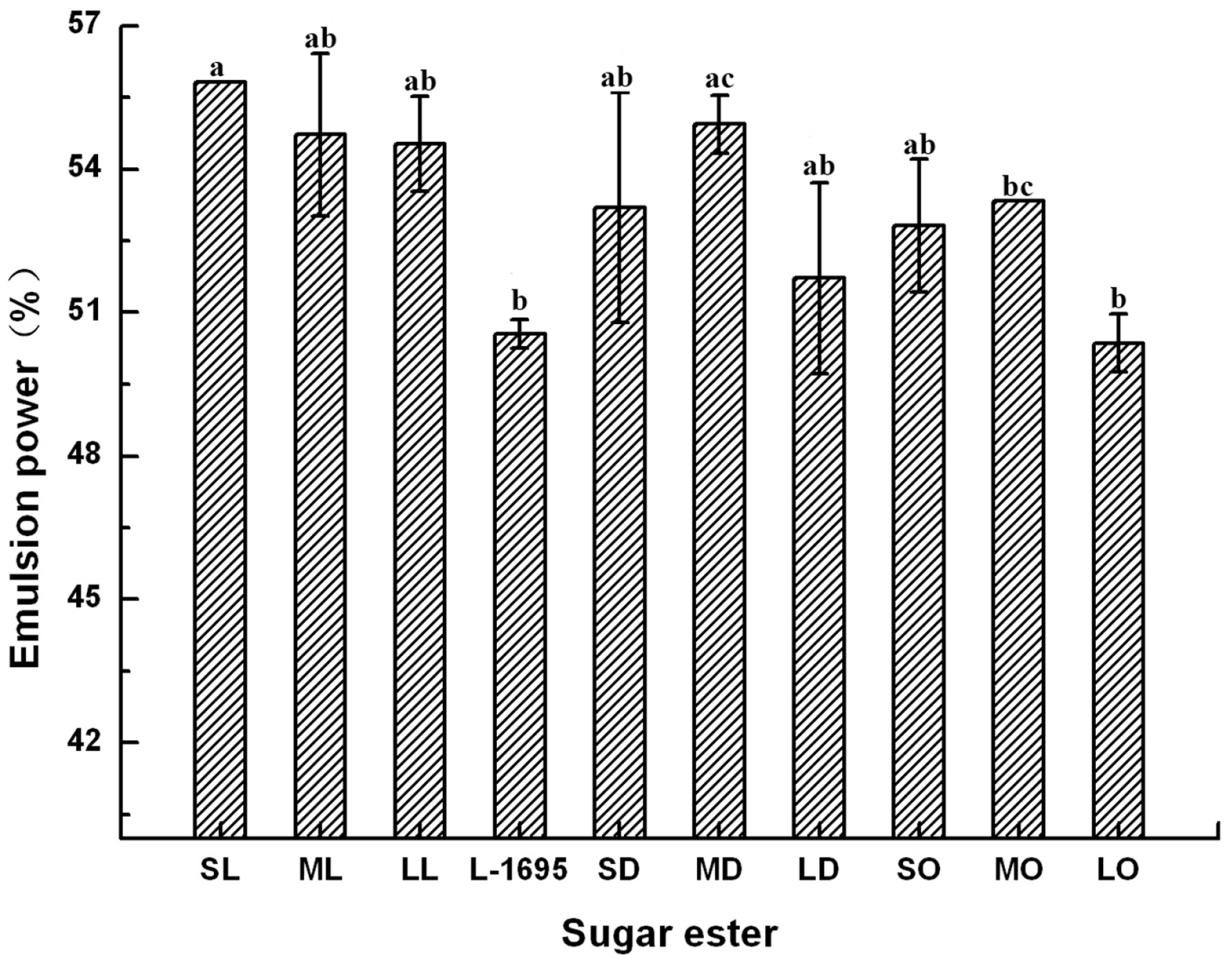
Emulsion power of different sugar esters at a concentration of 0.02% (w/v) in soybean oil–water system. Different letters represent significant difference (*p*<0.05).

Soybean oil–water system was established with a surfactant content of 0.02% (w/v) in the aqueous phase, in order to investigate the effect of the sugar esters on stabilizing emulsions. The emulsifying abilities of all esters exceeded 50%, indicating that disaccharide monoesters were efficient for emulsifying the soybean oil–water system, as shown in Fig. 4. This result was similar to that of previous work, which indicated that sorbitol monolaurate significantly increased the stability of oil-in-water emulsions, with only 5% separation of the phases after 48 h at 30°C [25]. The emulsifying ability of lauroyl monoesters was greater than that of L-1695, which could be explained in terms of the diester reducing the emulsion power [32]. The monoesters with longer carbon chains showed better emulsifying ability than those with shorter chains because the disaccharide monolaurates with lower value of HLB were more compatible with this soybean oil–water system.

**Figure 5:**
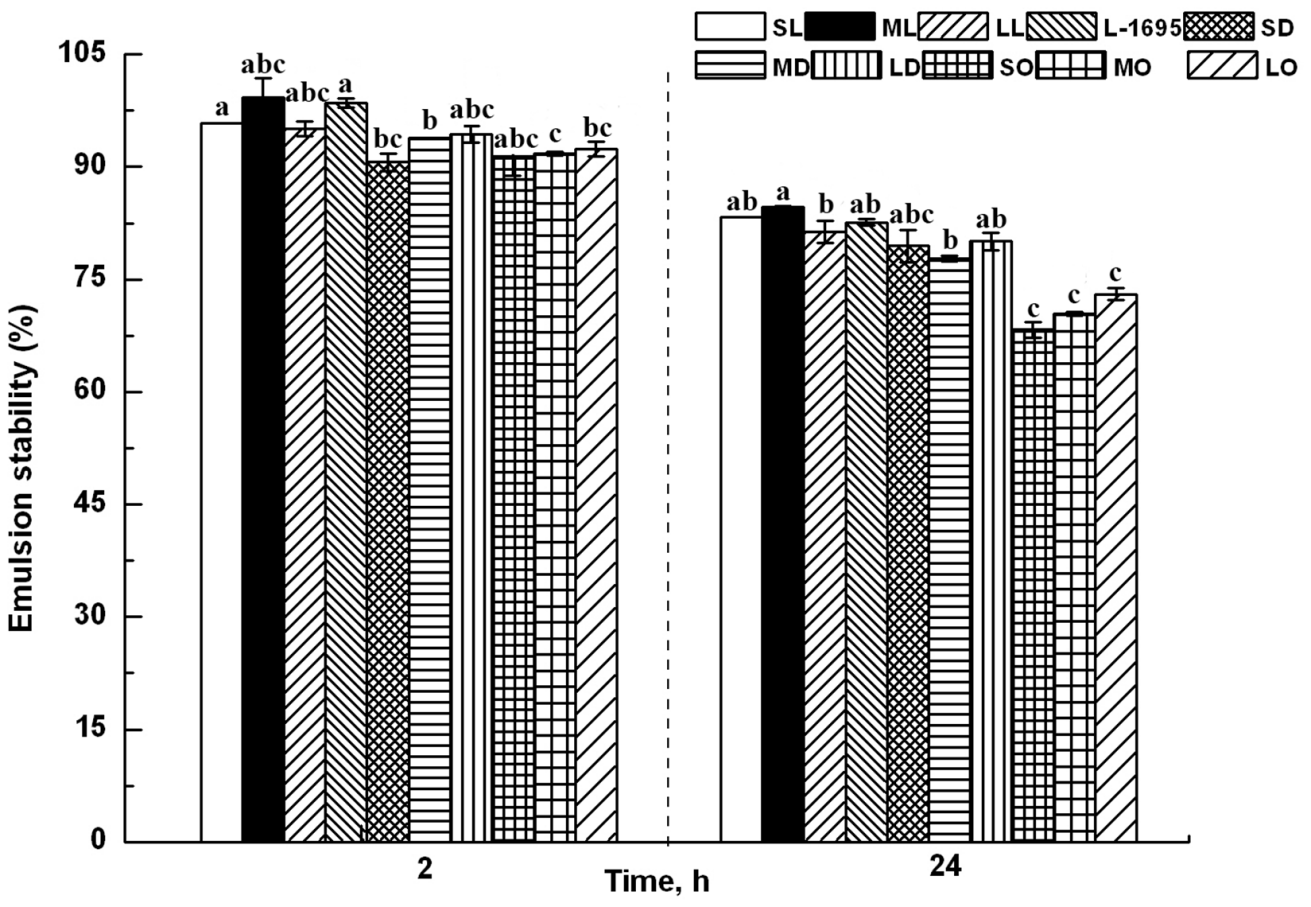
Emulsion stability of sugar esters in 0.02% (w/v) aqueous solution at 25°C. Different letters represent significant difference (*p*<0.05).

The emulsion stabilities versus time of the various esters are shown in Fig. 5. The stability of all sugar esters exceeded 90% from 30 min to 2 h, which means they have excellent emulsion properties. The stability decreased with an increase of standing time. The emulsion stability of the monocaprylate esters reduced the most with values below 75%, while the lauric acid esters showed the best stability. These results indicated that an increase in lipophilic chain length was needed, as expected, for increasing the emulsion stability of the medium-chain fatty acid monoesters.

### Antimicrobial properties of sugar esters

Sugar esters are primarily used as emulsifiers in the food industry. In recent years, the antifungal and antibacterial properties of sugar esters have been extensively investigated, these esters being widely used as canned beverage preservatives in Japan. Thus, most previous studies were focused on lauryl ester and commercial derivatives, and the test samples were complex mixtures of monoester, diester, etc., and containing different regioisomers. The effects of disaccharide core (sucrose, maltose, lactose), length of the fatty acid (caprylic, capric and lauric acid), degree of substitution (monoester and diester) and anomeric configuration (α- and β-ester) on antimicrobial properties were assessed comprehensively in this research.

Three common pathogens, *S. aureus* (Gram-positive bacterium), *E. coli* (Gram-negative bacterium) and *C. albicans* (yeast), were chosen to analyze the antimicrobial effect of the sugar esters. The OD values of blank negative control were not changed after 24 h or 48 h, and OD values of positive control were the highest in the experiments in order to obverse the growth of microorganisms. The MIC values of all the sugar esters against *E. coli* and *C. albicans* could not be measured at the concentration of 3000 µg/mL. Some researchers have reported that sucrose fatty acid esters could inhibit *E. coli*, [33], [34] but other researchers sucrose monolaurate had no antimicrobial activities against *E. coli* [5], [6]. The resistance was attributed to the cytoderm lipopolysaccharides and membrane lipids, which could screen out the fatty acid and prevent accumulation in the transport cell membrane. [35]–[37] Lauroyl glucose displayed MIC values>500 µg/mL for C. albicans and C. lipolytica [38]. Table 3 shows the antimicrobial properties of the sugar esters at a concentration of 3000 µg/mL. The sugar monodecanoate and sugar monooctanoate had better antibacterial activity against E. coli than sugar monolaurate, while the antimicrobial activity against C. albicans was the opposite. This phenomenon implied that acyl donor of sugar monoester was very important for antimicrobial activities, which might influence the physiological function.

**Table 3:** Screening of antimicrobial properties of a series of sugar monoesters and L-1695 at a concentration of 3000 µg/mL a.

Sugar ester	*E. coli*	*C. albicans*	Sugar ester	*E. coli*	*C. albicans*
**SL**	+	+	MD	++	−
**ML**	+	+	LD	+	−
**LL**	+	+	SO	++	−
**L-1695**	+	+	MO	++	+
**SD**	++	−	LO	++	−

a ++, inhibition >50%; +, inhibition between 30% and 50%; −, inhibition <30%.

The inhibitory effects against *S. aureus* are presented in Table 4. Sugar monoesters at a relatively low concentration were inhibitory to the growth of *S. aureus*, whereas *E. coli* was more resistant to their effects. The Gram-negative bacterium was more resistant to the inhibitory effects of the sugar esters because of membrane structure and difference in cell wall [35]–[37]. Similar findings were described when microorganisms were treated with fatty acids, glycerides and monosaccharide esters [38].

**Table 4:** MIC values of sugar esters for *S. aureus* and standards in TSB at 37°C for 24 h.

Sugar ester	MIC (µg/mL)	Sugar ester	MIC (µg/mL)
**SL**	250	MD	4000
**ML**	250	LD	4000
**LL**	500	SO	>4000
**L-1695**	500	MO	>4000
**SD**	4000	LO	>4000

Monolauroyl sucrose and monolauroyl maltose showed MIC values of 250 µg/mL against *S. aureus* compared to the value of 500 µg/mL of monolauroyl lactose, which indicated that different sugar group could affect the antimicrobial activities. These results corroborated previous findings showing that several monosaccharide esters could inhibit the growth of *Streptococcus mutans* with MIC values in the range 50–200 µg/mL [2]. Liu found that monolauroyl maltose and monolauroyl sucrose inhibited the growth of *Bacillus cereus*, *B. coagulans*, *B. subtilis*, *Geobacillus stearothermophilus*, *E. coli* and *S. aureus* at 0.09% bulk concentration [39]. Devulapalle et al. showed that 6-*O*-lauroylsucrose, 6′-*O*-lauroylmaltose and 6″*-O-*lauroylmaltotriose at 100 µg/mL could completely inhibit *Streptococcus sobrinus*, which is a mutant *Streptococcus* with a key role in the initiation of dental caries [4]. However, Ferrer et al. found that 6-*O*-lauroylsucrose and 6′-*O*-lauroylmaltose inhibited the growth of *Bacillus* sp. at a concentration of 800 µg/mL, but *S. aureus* could not be inhibited at 4000 µg/mL [6]. A different effect of the sugar esters was observed between the Gram-positive and Gram-negative bacteria. Overall, the antimicrobial activity of the sugar esters against the Gram-positive bacterium was greater than that against the Gram-negative bacterium and fungus. The Gram-negative bacterium was resistant to the inhibitory effects of the sugar esters.

The degree of esterification of lauroyl sucrose was crucial for antimicrobial activity. The commercial ester L-1695 is a mixture of different degrees of esterification of lauroyl sucrose with 80% of monoester and 20% of sucrose dilaurate. The MIC value of L-1695 was twice that of pure monolauroyl sucrose, from which could be inferred that diester could affect the antibacterial activity. Several researchers indicated that di- and tri- esters did not display antimicrobial activity, probably due to their low aqueous solubility [6], [38]. In this study, the synthetic sugar esters had a higher purity, higher than 90% compared to 80% for L-1695, which could illustrate that monoester was the major antibacterial component. The L-1695 inhibition of *S. aureus* at a concentration of 250 µg/mL was 88.2%.

The antimicrobial test of the sugar esters reflected the relationships between molecular structure and antibacterial activity. The length of fatty acid chain had a notable effect on antibacterial activity. The lauroyl monoesters showed best antimicrobial activities among medium chain fatty acid monoester against *S. aureus*, while caprylyl monoesters were the least active compounds tested, with comparatively negligible MIC value of >4000 µg/mL. For example, sucrose monoesters have the same hydrophilic group, but different hydrophobic groups. The order of increasing effectiveness of carbon chain length was C8<C10<C12, which could illustrate that the balance of hydrophilic groups and lipophilic groups played an important role in the inhibitory effect. This phenomenon leads to the speculation that sugar ester may be combined to the surface of the bacteria via acyl moieties to influence the physiological function.

Lauroyl maltose ester (α-ester) and lauroyl lactose ester (β-ester) are isomers, but their MIC values were quite different, which supported the results of Smith that the antimicrobial activities of monoesters were affected by conformation of carbohydrate itself [38]. Lauroyl maltose showed higher activity than lauroyl lactose against *S. aureus*, indicates that the anomeric configuration of the sugar could affect the antibacterial efficacy. Generally, the α-configuration compound is more effective than the β-configuration for the same carbohydrate, which was similar to our result [1]. However, Smith found a difference when the lauric ether anomers of methyl glucopyranosides were tested against *S. aureus*, with the β-configuration showing a higher activity [38].

## Conclusion

9 different sugar monoesters of three disaccharides with different carbon chain lengths (C8–C12), which synthesized by immobilized lipase (Lipozyme TLIM), have been studied with respect to their CMC and efficiency in reducing the surface tension of water. The CMC increased with decreasing carbon chain length, while caprate monoesters exhibited lower surface tension.

Foamability, foaming stability, oil–water emulsifying ability and emulsion stability of the monoesters were measured. The results indicated that the surface properties were affected by the carbon chain length, degree of esterification and hydrophilic groups. The laurate monoesters showed the best properties as a surfactant.

Monolauroyl sucrose and monolauroyl maltose showed the best antimicrobial activity with an MIC value of 250 µg/mL against *S. aureus*. However, *E. coli* and *C. albicans* could not be inhibited at a concentration of 3000 µg/mL indicating that the sugar monoesters were more effective against Gram-positive than Gram-negative bacteria. The antimicrobial activity was also influenced by the carbon chain length, degree of esterification and hydrophilic groups.

For the disaccharide medium-chain fatty acid monoesters, the length of the fatty acid chain (hydrophobic groups) is the most important factor affecting surface activity and antimicrobial activity, while the saccharide groups (hydrophilic groups) and degree of esterification are less important.

## Supporting Information

S1 Fig **Structure of sucrose monoester.**
(TIF)

S2 Fig **Structure of maltose monoester.**
(TIF)

S3 Fig **Structure of lactose monoester.**
(TIF)
