# Supplementary figures and images for: Comparative Study of Surface-Active Properties and Antimicrobial Activities of Disaccharide Monoesters
Source: PLoS One. 2014 Dec 22;9(12):e114845. doi: 10.1371/journal.pone.0114845 (PMC4273987; doi:10.1371/journal.pone.0114845)

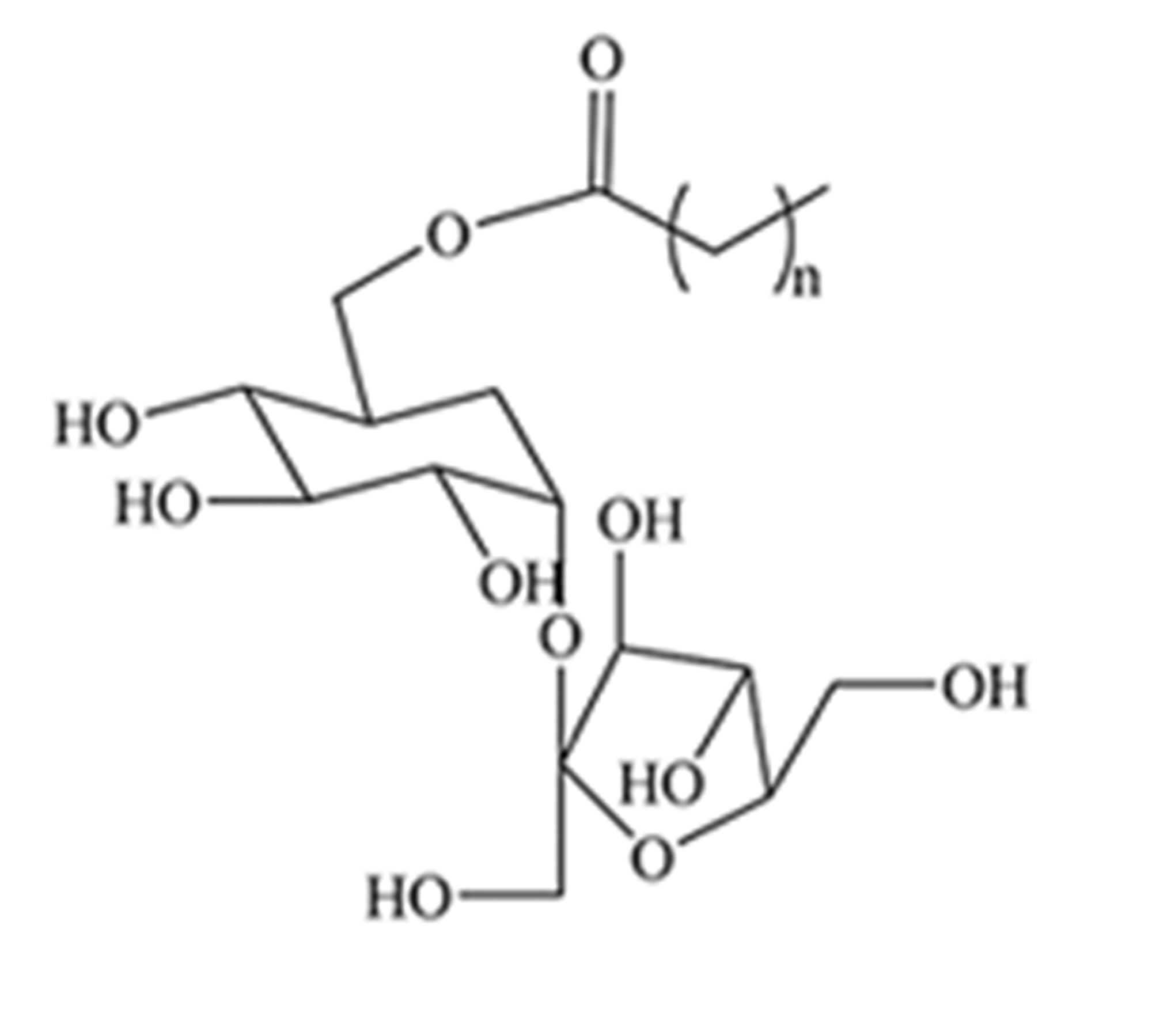

Supplement: S1 Fig — Structure of sucrose monoester. (TIF) [file pone.0114845.s001.tif]

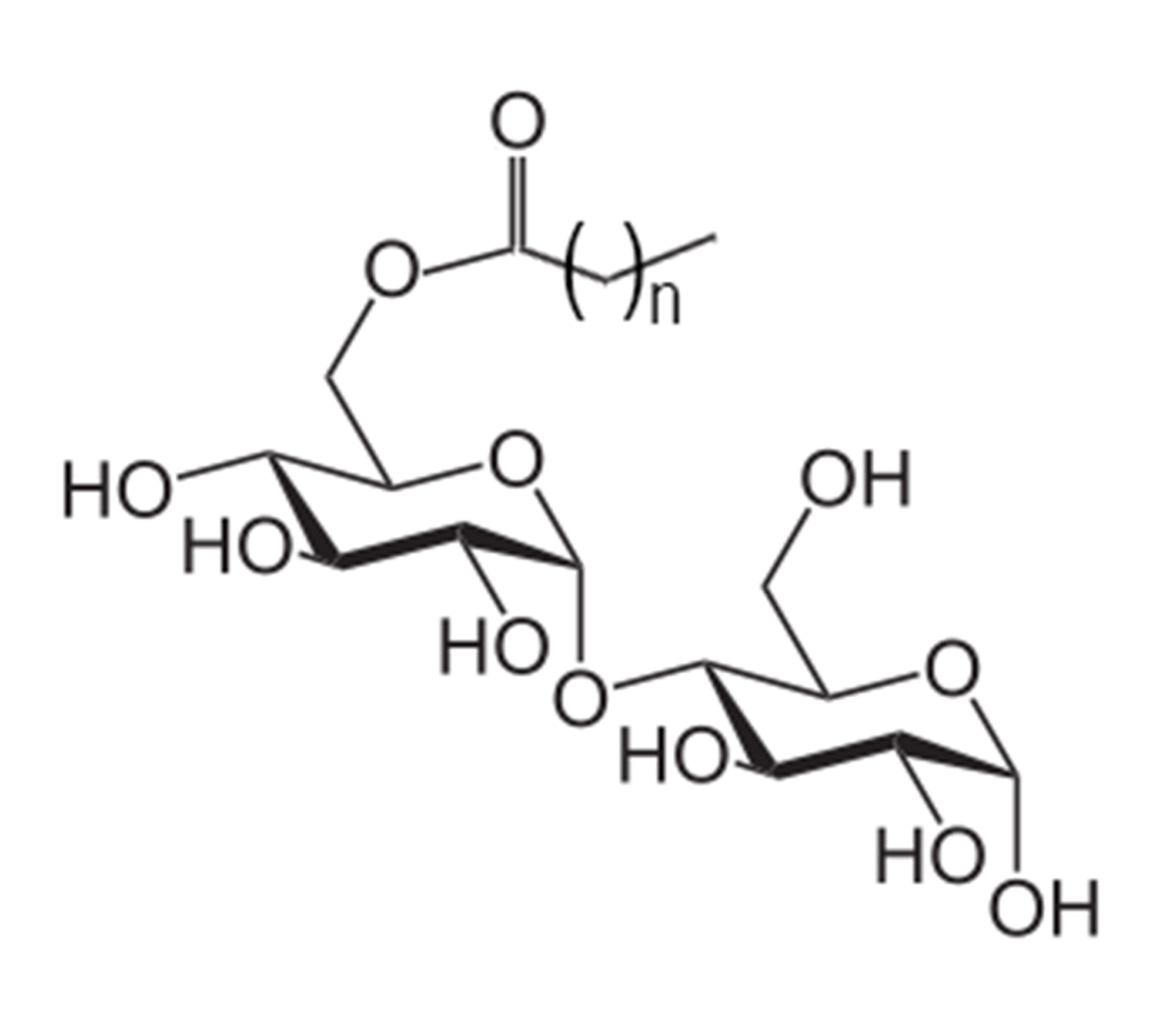

Supplement: S2 Fig — Structure of maltose monoester. (TIF) [file pone.0114845.s002.tif]

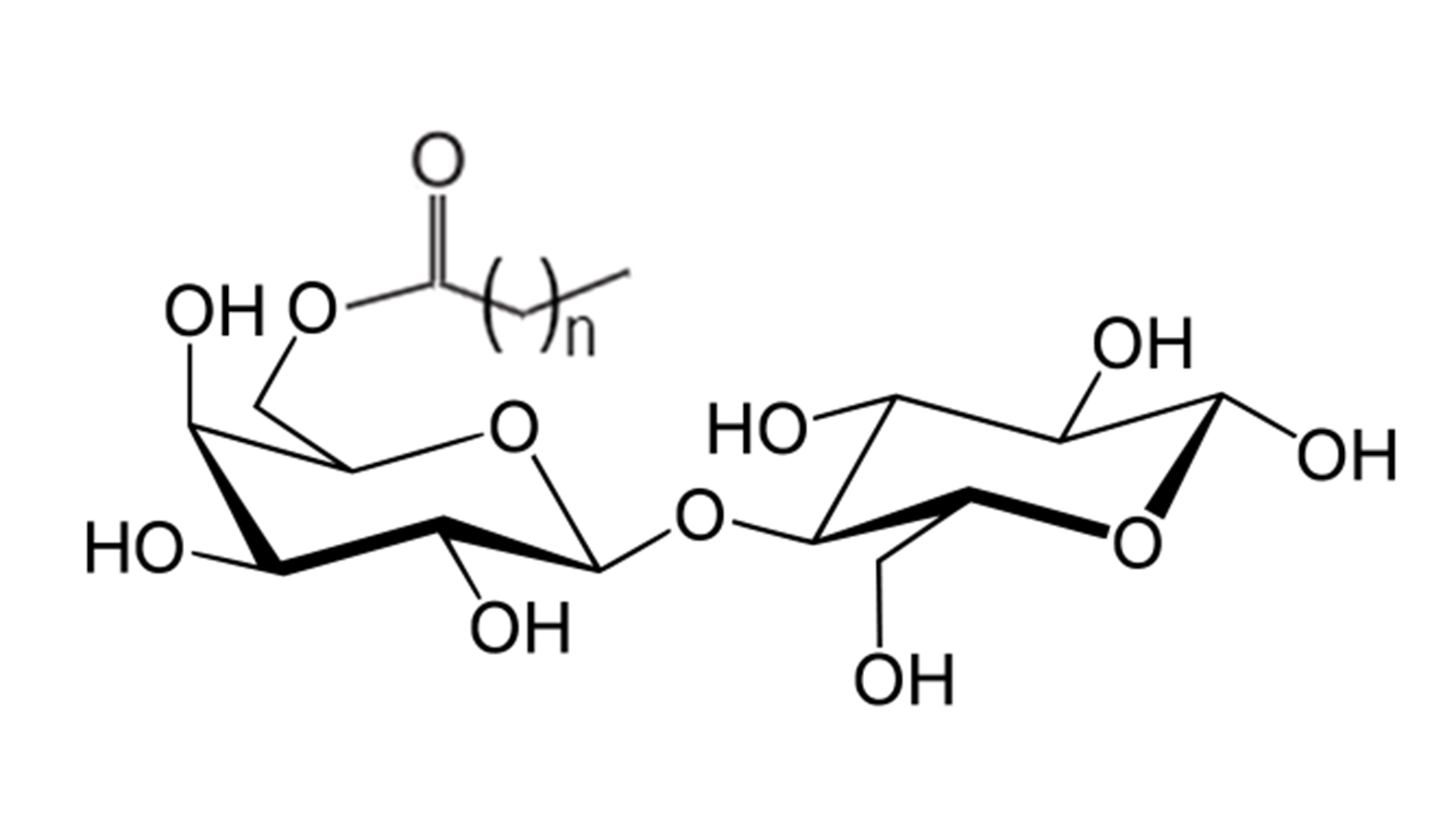

Supplement: S3 Fig — Structure of lactose monoester. (TIF) [file pone.0114845.s003.tif]
